# Cognitive Control Modulates Effects of Episodic Simulation on Delay Discounting in Aging

**DOI:** 10.3389/fnagi.2017.00058

**Published:** 2017-03-14

**Authors:** Laura K. Sasse, Jan Peters, Stefanie Brassen

**Affiliations:** ^1^Department of Systems Neuroscience, University Medical Center Hamburg-EppendorfHamburg, Germany; ^2^Department of Psychology, University of CologneCologne, Germany

**Keywords:** aging, impulsivity, delay discounting, episodic prospection, cognitive control

## Abstract

Enhancing prospective thinking by tagging the future with specific episodic events has been shown to reduce delay discounting in young age (“tag-effect”). So far, it is unclear whether such beneficial effect extends to old adulthood. Since the general ability of future thinking and cognitive control are crucial modulators of temporal discounting in young age, potential age-related decline in these functions might impact on the effect. We focused on this issue by combining functional magnetic resonance imaging (fMRI) with an established intertemporal choice task including episodic “tags” in healthy older participants. Future thinking ability was assessed using autobiographical interviews for future event simulations and a visual search task was applied to assess participants’ cognitive control ability. In contrast to previous data in young adults, the group of older participants did not benefit from tagging the future with episodic events. Older participants’ cognitive control function was directly associated with discounting rates in the episodic conditions: the less the older adults were able to focus their attention the less they benefited from the inclusion of episodic events. Consistent with this, imaging results revealed that: (a) subjective value (SV) signals in the hippocampus and the anterior cingulate cortex (ACC) as well as; (b) hippocampal-striatal coupling during the episodic condition were positively related to participants’ control capacity. Our findings highlight the critical role of executive functioning for the simultaneous integration of episodic information with future value computation in aging. Boosting delay gratification by including episodic tags might hence be limited in older individuals with pronounced decline in distraction control.

## Introduction

Delay discounting, the tendency to devaluate rewards as a function of time to their delivery, has been linked with harmful health behaviors (Chabris et al., 2008; Reimers et al., 2009) and is known to be increased among individuals with impulse control disorders (Bickel et al., 2014) and low levels of executive control (Shamosh et al., 2008; Bickel et al., 2011). Studies in younger adults have demonstrated that one critical modulator of individual discounting rates is the degree to which participants engage in episodic future thinking (Bromberg et al., 2015; Wiehler et al., 2015). Intriguingly, future thinking in the context of delay gratification can be boosted by combining delays with specific future events (Peters and Büchel, 2010; Benoit et al., 2011; Palombo et al., 2015; Sasse et al., 2015). On a behavioral level, such episodic manipulation typically leads to reduced discounting rates possibly due to the facilitated anticipation of future time-points by pre-experiencing a specific future event (“tag-effect”). In this line, neural findings show an increased engagement of episodic memory circuits and a heightened integration of such episodic signals into value signals by prefrontal-limbic reward circuits when individuals evaluate future delays combined with episodic events (Peters and Büchel, 2010; Sasse et al., 2015).

It is yet unclear whether such a tag-effect prevails until old age. Differences might be expected due to age-related changes in: (i) future thinking ability (Addis et al., 2008, 2011); and (ii) executive functioning—two important functions for the controlled integration of episodic information with value processing (Samanez-Larkin and Knutson, 2015). Along those lines, older adults have shown deficits in the detailed imagination of future episodic events (Schacter et al., 2013) and an increased susceptibility to memory distortions (Gerlach et al., 2014), even though there is typically high variability in age-related changes in episodic processing (Nyberg et al., 2012). Specifically, when remembering past and imagining future events, younger and older adults engage a parieto-fronto-temporal network including the hippocampus, precuneus und prefrontal cortex (Viard et al., 2011; Schacter et al., 2012, 2013). Yet, individual differences may occur due to age-specific decline in these regions (Addis et al., 2011; Persson et al., 2011; Pudas et al., 2014).

A critical impact of executive functioning on the tag-effect in aging may be hypothesized based on observed impairments in value-based decision making under conditions of high cognitive load (Lighthall et al., 2014) and deficits when learned information needed to be integrated in the decision-process (Mata et al., 2011) in older adults. According to the inhibitory control hypothesis (Hasher and Zacks, 1988), such deficits in multidimensional information processing might primarily result from age-related impairments in controlling interfering information. Increased anticipation of future options through episodic simulation, as evident during the “tag-effect”, requires the simultaneous processing and integration of prospection and valuation signals. Older adults’ ability of executive control might thus be a critical determinant of beneficial effects from future thinking on delay gratification in aging.

In the present study, we investigated whether healthy older participants benefit from episodic stimulation during delay discounting, i.e., show enhanced delay gratification when a future reward is combined with an episodic event (“tag”) as previously demonstrated in young adults (Peters and Büchel, 2010; Sasse et al., 2015). In this context, we were interested in the impact of participants’ future thinking and executive functioning ability on the occurrence of a neurobehavioral tag-effect. Specifically, we tested whether older participants demonstrate significantly lower discounting rates when future delays are combined with episodic events. Moreover, we investigated whether better general memory function, reported detailedness of imagined future events and stronger engagement of the neural episodic memory network during episodic conditions is positively related to the occurrence of the tag-effect. Finally, we tested the hypothesis that older participants with higher cognitive control ability are better able to benefit from integrating episodic information into value computation which should be reflected by a positive correlation of individuals’ control ability with the tag-effect paralleled by the activation of memory-reward key regions.

To this end, we combined functional magnetic resonance imaging (fMRI) with an established intertemporal choice paradigm (Sasse et al., 2015) that allows for a systematic investigation of the influence of episodic simulation on delay discounting. In this paradigm, some delays are combined with a specific future event (i.e., meeting different people in a café) and compared to trials without episodic prospection. The task was followed by an autobiographical interview. General episodic memory ability was assessed using a standardized verbal learning memory task. In addition, executive control ability was measured using a well-established visual search task (Theeuwes and Burger, 1998; Costello et al., 2010) which has previously demonstrated a valid applicability in older adults (Sasse et al., 2014). Based on neural findings in younger adults, we focused our factorial and functional connectivity analyses on networks engaged in episodic prospection and reward integration.

## Materials and Methods

### Participants

Twenty-two healthy older adults (*M* = 66.55; *SD* = 4.02; 60–74 years; 9 men) participated in the present study. Participants were recruited from an existing database and gave written informed consent before their participation. It was ensured that all participants had no present or previous neurological or psychiatric disorders like depression or dementia and successfully completed the neuropsychological battery of the Consortium to Establish a Registry for Alzheimer’s Disease (CERAD) including the Mini-Mental State Examination (MMSE, all participants <28). Participants were financially compensated with 10 Euros per hour. In addition, one chosen reward from the delay discounting task was randomly selected and paid out with the respective delay. The local ethics committee (Aerztekammer Hamburg) approved the study. All participants gave written informed consent before participation. No vulnerable populations were involved.

### Study Design of the Discounting Task

**Figure 1:**
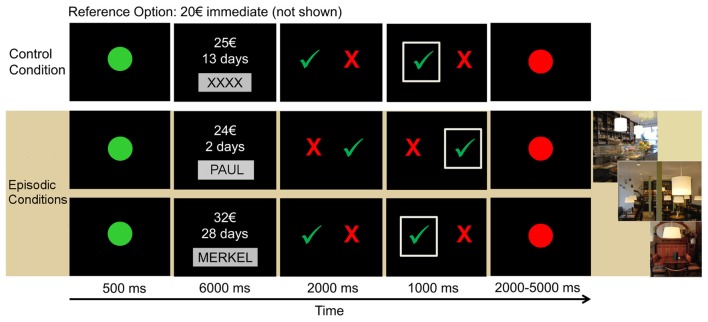
**Outline of the paradigm.** Each trial started with a green dot, signaling the start of the trial. Then, the delayed reward option was presented for 6 s and participants had to either imagine the event in the café (episodic conditions) or not imagine anything in the control condition. Subsequently, participants had to indicate their choice by selecting the red cross for the immediate reward (20€ that were not shown) or the green check mark for the delayed reward option.

The design and procedure of the fMRI discounting task have previously been applied in a group of younger adults (Sasse et al., 2015; Figure 1). The experiment consisted of three conditions presented in six blocks (two blocks per condition). Two of these six blocks served as the control condition, which involved standard delay discounting without episodic prospection, while the other four blocks were assigned to two episodic tag conditions, requiring participants to imagine meeting a person in a café for the day of delayed reward delivery.

In a preparatory interview, participants were asked to identify four persons, two familiar and two unfamiliar persons, they would like to meet in the future. These persons were identified using a standardized interview (adopted from Carstensen and Fredrickson, 1998). For the identification of the familiar social partners, participants had to imagine moving abroad on their own and to appoint familiar persons with whom they would like to spend the last hours before their departure. To identify the famous, novel partners, participants imagined conducting an interview for a newspaper with persons of public interest whom they had never met in person. This procedure was used for two reasons: first, the inclusion of different persons from different backgrounds should limit habituation effects during the paradigm. Second, it makes it possible to investigate the impact of emotional closeness on discounting behavior, which has previously been identified as a key modulator of choice behavior in older age (Fredrickson and Carstensen, 1990; Fung and Carstensen, 2004).

During each trial, participants were required to choose between a fixed immediate reward option of 20€ (which was not shown on the screen) and a larger but delayed amount. During the episodic conditions, this delayed reward option was presented together with the name of the social partner with whom they had to imagine a meeting in a café for the date of the delayed reward delivery. In the control condition, delayed options were presented together with placeholder strings (“XXXX” or “YYYY”) and participants were explicitly instructed to refrain from imagery.

In each block, participants viewed 36 trials involving six different delays that were randomly drawn from one of two sets [1, 2], [6, 7], [13, 15], [28, 32], [85, 95], [170, 190]. Next, the six delays were paired with six monetary amounts, ranging from 20.5€ to 79.5€. While minimum and maximum amount for each delay were close to the extreme values for each participant, values varied individually between these extremes for each participant. More specifically, these values were participant-specific constructed based on a computer-based delay-discounting procedure participants completed on the date prior to scanning. Choice data from this pretest were fitted using a hyperbolic discounting function of the form

$$SV\;=\;\frac{A}{\left(1+kD\right)}$$

to estimate the individual discount rate for a reward of 20€ (SV, subjective value; A, amount of the delayed reward; D, delay in days; k, discount rate; Mazur, 1987). The discount rate was then used to calculate indifference amounts for six delays for each participant (i.e., points where the participant valued the immediate and the delayed reward as equivalent). Subsequently, the six delays were paired with amounts that lied equally above and below the respective indifference point. This procedure has been applied in previous experiments in our lab to ensure that participants would choose the delayed option in 50% of the trials (see Sasse et al., 2015).

In order to avoid sequence effects, the presentation of the three conditions (control, familiar event, unfamiliar event) was randomized but the two blocks of each condition were always presented successively. Between blocks, participants were given a short break to relax. Participants were trained on the task and familiarized with five images depicting scenes of a typical café before the experiment. After the end of the task and without being scanned, participants remained lying in the scanner for approximately 10 min for an interview about the richness of their imagination for the four episodic events (two familiar, two unfamiliar). During this interview, participants were asked to describe their imaginations for each of the four events as detailed as possible. Answers were recorded to be later transliterated. Outside the scanner, participants were asked to rate the emotionality they associated with the four partners as well as their motivation to meet the partners for each event on scales ranging from 1 to 7.

In addition, participants’ general memory capacity was assessed on a separate day prior to scanning via the verbal learning and memory test (VLMT; Helmstaedter et al., 2001). The VLMT involves verbal word list learning (in five consecutive trials) and we used the sum of recalled words across all five trials as an indicator of individual memory capacity.

### Attentional Control Task

On the day prior to scanning, participants performed a visual search task (Theeuwes and Burger, 1998; Costello et al., 2010). Application and analysis was based on our previous work where older participants’ ability to control attention during highly salient distraction explained substantial variance during emotion processing (Sasse et al., 2014).

**Figure 2:**
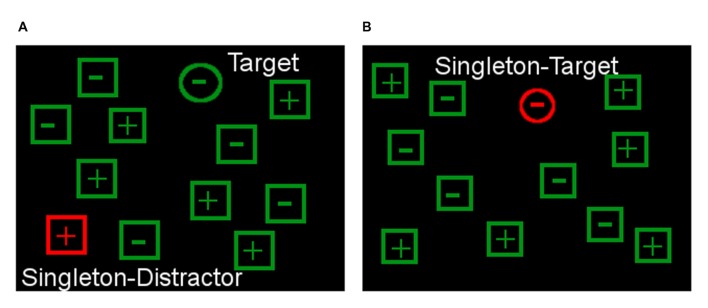
**Attentional control paradigm.** The task is to respond to the symbol depicted in the target shape (circle) and ignore the singleton distractor **(A)**. Demands on flexible attentional control can be raised by including trials in which the singleton can become the target **(B)** so that it cannot be blanked out from the start.

Specifically, participants had to indicate by button press as quickly (<3 s and accurately as possible whether a target circle included a “+” or “−”. The target was surrounded by non-targets differing in shape (squares) from the target (Figure 2). The task included two conditions: in condition one, the target (green circle) was surrounded by a different amount of green distractors which sometimes (50% of the trials) included one red square (= singleton distractor). Participants could accomplish the task in this condition without an actual need of flexible control of attention by strategically focusing on the shape dimension while blanking out the color dimension (Costello et al., 2010). In condition two, the target was colored in red (red circle, = singleton target) in 8% of the trials. Now, the formerly inhibited red color dimension became highly relevant which required a flexible use of attention control (Costello et al., 2010). The two conditions were presented in separate blocks (each condition *n* = 4 blocks with 48 trials) and the type of condition was announced before the start of each block.

We then computed an index score of participants’ ability to flexibly control attention that was adjusted for more strategic forms of attentional control (Sasse et al., 2014). Specifically, increases in reaction time (RT) due to the colored distractor in condition one (= no flexible control required) were subtracted from increases in RT due to colored distractor in condition two (= flexible control condition). Consequently, a higher singleton score implied less flexible attentional control.

### Data Acquisition

Presentation software (Neurobehavioral Systems©) was used for stimulus presentation and recording. fMRI data were acquired on a 3 tesla system (Magnetom Trio, Siemens) equipped with a 32-channel head coil. Each volume comprised 41 transversal slices (2 mm thickness, 1 mm gap, TR = 2460 ms, TE = 25 ms, FOV = 216 × 216 mm^2^, in-plane resolution 2 × 2 mm^2^, GRAPPA factor 2). High-resolution anatomical MR images were acquired after functional imaging using a T1-weighted MPRAGE sequence (1 × 1 × 1 mm).

### Behavioral Data Analysis

For the behavioral data analysis, individual choice data were fitted using Maximum Likelihood Estimation (MLE) by combining the aforementioned hyperbolic discounting function with softmax action selection (Peters et al., 2012) separately for each experimental condition in Matlab (Mathworks©). This yielded two free parameters per condition, the hyperbolic discounting constant *k*, where higher values reflect greater impatience, and the inverse temperature parameter *β* of the softmax choice function, where greater values reflect more decision noise. Following Sasse et al. (2015), a square-root transformation was applied to the resulting *k* parameters prior to the analyses, accounting for their skewed distributions. Table 1 depicts model characteristics, such as medians and Inter-Quartile-Ranges (IQRs) of the absolute single-subject maximum likelihood parameter estimates and RT data. All statistical analyses included gender as covariate of no interest to account for unequal distributions of men and women.

**Table 1:** **Model Parameters**.

	*k*		*β*		RT	
	Median	IQR	Median	IQR	Median	IQR
Control	0.085	0.18	2.15	4.72	861.84	399.99
Tag	0.082	0.19	3.19	8.20	909.59	375.99
Familiar	0.089	0.20	3.14	8.25	911.50	385.75
Unfamiliar	0.080	0.19	2.72	10.73	929.93	438.59

*For each of the conditions, medians and inter-quartile ranges (IQR) are reported for the model estimates of the discounting parameter (k) and temperature parameter (ß) as well as for the reaction times (RT)*.

Descriptions of the imagination ratings for the four events were analyzed with respect to the level of episodic richness using a rating procedure based on the Autobiographical Interview (Levine et al., 2002). Details were categorized as internal (episodic information relating to the given future event) or external (non-episodic information). Internal details were categorized further into one of five categories adapted from Levine et al. (2002): time, place, perceptual, emotions/thoughts and event details. External details comprised semantic details, repetitions and other metacognitive statements, but were combined into a single score since there were only very few external details. A second independent rater coded details into the same categories, yielding a reliability between the raters of cronbach’s alpha = 0.81 for internal details and cronbach’s alpha = 0.81 for external details.

### fMRI Data Analysis

fMRI data were pre-processed and analyzed using statistical parametric mapping (SPM8; Welcome Department of Imaging Neuroscience, London, UK).

Functional data were corrected for slice timing before being realigned and unwarped. Next, the individual structural T1 image was coregistered to the mean functional image generated during realignment. Coregistered T1 images were then segmented using the “New Segment” routine in SPM8. Resulting tissue-class images for gray and white matter were subsequently used for spatial normalization of the functional images using the DARTEL toolbox. Data were smoothed with a 6-mm full-width at half maximum (FWHM) isotropic Gaussian kernel.

Using the general linear model (GLM) denoise toolbox for Matlab (Kay et al., 2013), the data were then “denoised” by deriving regressors from voxels whose activation was unrelated to the manipulation of the experimental paradigm and entering these regressors in a GLM analysis. In this GLM, sustained activation during the presentation of the delayed option (i.e., from option onset until button press) was modeled by boxcar regressors that were convolved with the canonical hemodynamic response function. Condition-specific *k*-parameters from the scanning session were used for the calculation of the SV each delayed option (via the hyperbolic formula) and included as a parametric regressor in the GLM.

For each subject, contrast images for the two conditions (control/episodic) and for the respective SV regressor were constructed. These contrast images were passed to the second level where group contrasts were computed using one-sample *t*-tests and regression analyses on the single-subject contrasts. Regression analysis was applied to investigate correlations between functional brain patterns and cognitive control capacity measured via the Singleton score.

Coupling analyses were performed by extracting the deconvolved time courses from the seed region for each condition (block) separately. Coupling patterns of each condition were then directly compared with each other to assess the impact of episodic modulation (episodic vs. control).

We performed whole brain corrections for multiple comparisons at the cluster level using a cluster-threshold of FWE < 0.05 (cluster forming threshold *p* < 0.005 uncorrected). Small volume corrections for multiple comparisons (SVC) were performed for anatomical masks of the hippocampus, (Harvard Oxford atlas, probability threshold of 50%) and for the ventral striatum (8 mm spheres centered on *x, y, z*: +/− 14, 8, −8 mm (O’Doherty et al., 2004; Yacubian et al., 2006). In addition, we defined 10 mm spheres around the coordinates of the episodic prospection network implicated in the tag-effect with the same paradigm in younger adults (Sasse et al., 2015), including the ventromedial prefrontal cortex (vmPFC; *x, y, z*: −6, 58, −6 mm), the posterior cingulate cortex/precuneus (*x, y, z*: −4, −52, 36 mm) and the lateral parietal cortex (*x, y, z*: −50, −72, 28 mm). The threshold of small volume corrections (SVC) was set to *p* < 0.05 corrected for multiple comparisons using the family-wise error rate (FWE).

## Results

### *Post hoc* Ratings and Singleton Scores

*Post hoc* interviews revealed that the motivation to meet the familiar (*M* = 6.21, *SD* = 1.02) and unfamiliar social partners (*M* = 5.82, *SD* = 1.41) did not differ significantly, *p* > 0.28. As expected, familiar partners (*M* = 6.26, *SD* = 0.54) were rated as significantly higher on emotional closeness than the unfamiliar partners (*M* = 2.93, *SD* = 1.08), *t*_(21)_ = 11.91, *p* < 0.001. Analysis of the post-scan Autobiographical Interview indicated that participants imagined familiar and unfamiliar events with similar amounts of internal (*t*_(21)_ = 1.25, *p* > 0.22) and external details (*t*_(21)_ = 0.89, *p* > 0.38; Table 2).

**Table 2:** **Level of detail and episodic richness of simulations across future event scenarios**.

	Familiar		Unfamiliar	
	*M*	*SD*	*M*	*SD*
Internal details	6.55	5.86	5.86	4.86
Event details	2.23	3.10	2.09	2.60
Perceptual details	1.55	1.85	0.82	1.05
Place details	1.32	1.09	1.14	0.94
Emotion/thought details	1.45	1.34	1.82	1.82
External details	0.86	1.08	0.68	1.04

*Means (M) and standard deviations (SD) are reported for the amount of details imagined for familiar and unfamiliar events with further divisions into subcategories of internal detail categories. Due to a low number of semantic details, repetitions and other metacognitive statements, we combined them into a single external detail score (see e.g., Addis et al., 2009). Time details were not reported (due to the concrete time reference of the event) and are therefore not listed in the table*.

For explorative reasons, we compared reported levels of imagination richness from the older participants to the scores obtained in our previously published study with younger adults (Sasse et al., 2015), who performed the identical paradigm. We found significantly fewer reported internal details for both familiar (*T*_(43)_ = 2.6, *p* < 0.05) and unfamiliar (*T*_(43)_ = 3.03, *p* < 0.01) events in old compared to younger adults.

In the singleton task, only few trials had to be discarded from the analysis due to errors or missing responses (*M* = 4.52%, *SD* = 3.61%). The mean distraction score was 71.34, *SD* = 89.29. The mean memory score of the VLMT was 47.5, *SD* = 10.34.

### Behavioral Results

#### Discounting Rates

**Figure 3:**
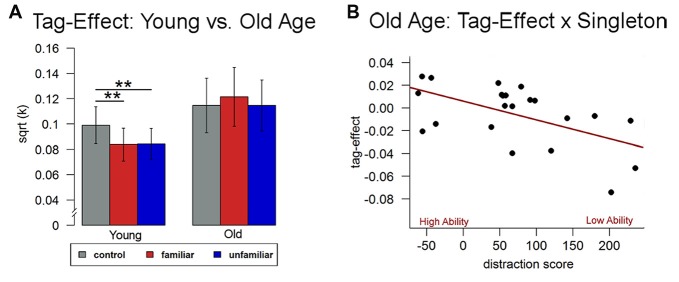
**Behavioral data. (A)** In comparison to younger adults (Sasse et al., 2015), discounting behavior did not significantly differ between the two episodic conditions and the control condition in older adults. **(B)** Differences between the two conditions in older age were related to individual differences in attentional control ability (singleton score). **p < 0.05.

Using a repeated measures analysis of variance (ANOVA) including the three conditions, we found no significant condition effect in older participants (*F*_(2,42)_ = 1.51, *p* > 0.32), i.e., there was no significant difference in discounting behavior in conditions with vs. without episodic tags. To further explore and validate this null-finding, we compared current discounting data in old adults with behavioral data from our recently conducted independent study in young adults (*N* = 23; mean age = 24.96 years; Sasse et al., 2015). Results showed a significant condition by group interaction, (*F*_(2,86)_ = 4.91, *p* < 0.05) and no main effects of group (*F*_(1,43)_ = 1.52, *p* > 0.22) or condition (*F*_(2,86)_ = 1.33, *p* > 0.26). The interaction was driven by a significant effect of the experimental manipulation on discounting rates only in the young subjects (*F*_(2,44)_ = 5.83, *p* < 0.01), thus confirming the lack of a tag-effect in older adults (Figure 3A).

#### Interactions with Episodic Thinking

Next, we tested whether discounting rates from the episodic conditions were related to older participants’ general memory ability (VLMT), level of imagined details (Autobiographical Interview) and emotional closeness of the imagined events. Here, we found no significant correlation, neither for the overall episodic discounting rate nor for the discounting rates in the episodic sub-conditions (all *p* > 0.48).

Please note that since none of the previous analyses revealed any differences between the two episodic sub-conditions, we concentrated all following analyses on the overall tag-condition.

#### Interactions with Cognitive Control Ability

We then analyzed whether participants’ individual control ability was related to discounting behavior. While there was no significant correlation with the discounting rate from the control condition (*p* > 0.11), we found a significant relationship between the singleton score and differences in discounting rates between the episodic and the control condition (*r* = −0.67, *p* < 0.05, see Figure 3B) indicating that the better participants’ control ability, the more their discounting rates were reduced in the episodic compared to the control condition.

### fMRI Data

#### Task-Related Activation Patterns

**Figure 4:**
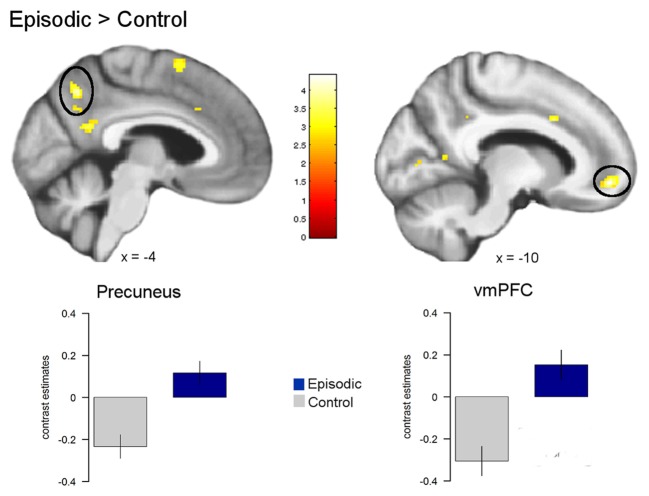
**Activation differences between episodic and control conditions.** Greater activation for the episodic conditions compared to the control condition was observed in the left ventromedial prefrontal cortex (vmPFC) and the left precuneus (all *p* < 0.05 FWE). Activations are overlaid on the mean structural image of all participants (display threshold *p* < 0.005 uncorrected).

First, general effects of episodic prospection (i.e., condition effects) on brain activity were analyzed. Here, we observed a significant increase in the BOLD signal across both episodic conditions compared to the control condition in the left vmPFC (−10, 52, −10, *z* = 3.97, *p* < 0.05 FWE) and the left precuneus (−4, −60, 42, *z* = 3.76* p* < 0.05 FWE). In line with previous studies on episodic prospection (Peters and Büchel, 2010; Benoit and Schacter, 2015), we also observed a cluster in more inferior posterior midline regions, i.e., the retrosplenial/posterior cingulate cortex (see Figure 4). However, this result did not survive our FWE correction procedure (−4, −54, 20, *z* = 3.40, *p* < 0.001 uncorrected).

In the next analyses, we focused on brain activity modulated by the SV of each trial (i.e., parametric effects). Across all conditions, there was a significant modulation by SV in the vmPFC, the orbitofrontal cortex, the posterior cingulate cortex and the bilateral lateral parietal cortex (Table 3). This modulation of SV did not significantly differ between the episodic and the control condition.

**Table 3:** **Regions in which the BOLD signal was significantly modulated by subjective value (SV) across all conditions**.

Brain Region	Side	MNI (peak)			Cluster size	*Z*-Score
		*x*	*y*	*z*		
vmPFC	b	0	54	−8	1768	4.71
Orbitofrontal cortex	l	−26	38	−10	370	5.10
Insula	l	−38	4	−2	1214	4.80
Precentral gyrus	r	56	2	36	380	4.36
Middle temporal gyrus	l	−66	−32	−2	429	4.76
Posterior cingulate cortex	l	−8	−34	48	2664	5.39
Lateral parietal cortex	r	58	−44	26	792	4.39
	l	−54	−56	22	1937	5.73

*MNI coordinates and z values are reported for peak voxels and local maxima within each cluster. All *p* < 0.05 family-wise error rate (FWE). l, left; r, right; b, bilateral*.

#### Modulation by Attentional Control Ability

**Figure 5:**
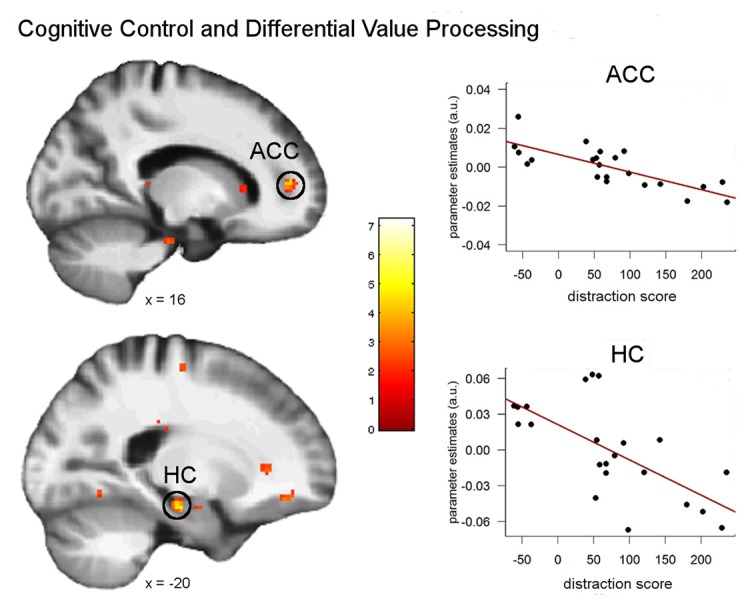
**Correlations between attentional control ability and neural modulation by subjective value (SV; parametric analysis) for the episodic compared to the control condition.** Significant correlations were found in the right anterior cingulate cortex (ACC) and the left hippocampus (all *p* < 0.05 FWE). Plots show the separate correlations in the peak voxels of the ACC and hippocampus. Activations are overlaid on the mean structural image of all participants (display threshold *p* < 0.005 uncorrected).

Subsequent analyses aimed at investigating whether our finding of a significant behavioral impact of attention control on episodic discounting rates is mirrored by an effect on episodic neuro-circuits and/or neural valuation signals. For this reason, we analyzed the potential impact on neural valuation by entering the singleton score as covariate into an analysis that compared parametric modulation by SVs between tag and control conditions (tag × SV < control × SV). This analysis revealed significant correlations in the right anterior cingulate cortex (ACC; 18, 46, 4, *z* = 4.95, *p* < 0.05 FWE), the left hippocampus (−20, −18, −18, *z* = 3.77, *p* < 0.05 FWE) and the left postcentral gyrus (−38, −24, 42, *z* = 4.06, *p* < 0.05 FWE; Figure 5), i.e., the higher the control ability, the stronger was the value signal in the tag compared to the control condition in these brain regions.

**Figure 6:**
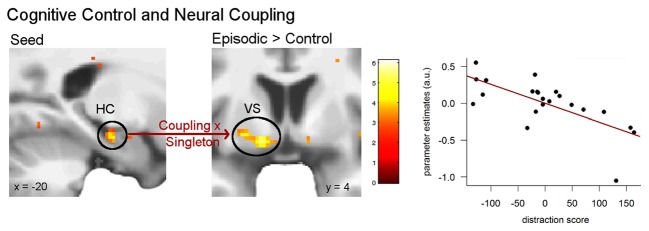
**Correlation between attentional control ability and hippocampal coupling.** Individual control ability (singleton score) was related to functional coupling between the left hippocampus and the left ventral striatum in the episodic compared to the control condition (*p* < 0.05 FWE). The plot demonstrates the correlation in the peak voxel of the left ventral striatum. Activations are overlaid on the mean structural image of all participants (display threshold *p* < 0.005 uncorrected).

Based on behavioral and neural findings, we were interested in the direct impact of control ability on the integration of episodic details with value coding. To this end, we analyzed whether coupling between the hippocampus (seed voxel from the above result: −20, −18, −18) and valuation circuits would differ in the episodic compared to the control condition depending on participants’ control ability. Regression analysis including the singleton score revealed a significant positive correlation with the left ventral striatum (−14, 4, −12, *z* = 4.01, *p* < 0.01 FWE; Figure 6), indicating that coupling between the hippocampus and the ventral striatum was greater for elderly people with higher control ability in the episodic compared with the control condition. Figure 6 shows an outlier in the data. Yet, after removing it from the analysis, the observed correlation remained significant.

## Discussion

We investigated the impact of episodic simulation on discounting behavior (“tag-effect”) in older adults and whether this effect is modulated by episodic prospection and/or executive control ability. To this aim, older adults with varying degrees of attentional control ability were examined with fMRI while performing an intertemporal choice task that included conditions in which the future delay was combined with an episodic event. In contrast to previous findings in young adults, the experimental induction of episodic prospection did not reduce discounting behavior in the older sample. However, a significant amount of heterogeneity in episodic discounting rates could be explained by older participants’ cognitive control ability. Specifically, results indicated that the lower older adults’ cognitive control ability, the less their discounting rate was decreased in the context of enhanced episodic simulation. Neuroimaging findings highlight two mechanisms underlying this result: higher cognitive control was related to: (a) stronger SV signals in the hippocampus and the ACC; and (b) tighter neural coupling between hippocampus and ventral striatum in the episodic compared to the control condition. There were no correlations between discounting behavior and memory capacity. Furthermore, participants engaged a well-defined network of episodic neuro-circuits in conditions including an episodic tag. These findings rather argue against the notion that general memory ability was a significant modulator of the tag-effect in this context.

In line with other studies (Green et al., 1996; Chao et al., 2009; Roalf et al., 2011; Samanez-Larkin et al., 2011; Rieger and Mata, 2015), delay discounting rates in the non-episodic condition observed in the present study did not differ from those previously observed in young adults (Sasse et al., 2015). However, in contrast to published data in young participants (Peters and Büchel, 2010; Sasse et al., 2015), older adults did not demonstrate reduced discounting rates when future options were combined with episodic tags. In our previous imaging studies on the tag-effect in younger age, successful integration of episodic information with value computation was related to increased SV signals in the hippocampus and the ACC (Peters and Büchel, 2010; Sasse et al., 2015). The hippocampus is thereby thought to modulate value computation by providing episodic prospection of decision outcomes, leading to a reduction in temporal discounting. In a similar vein, episodic signals from the hippocampus have been found to modulate reward-based decision-making via connections to the ventral striatum in animals (Johnson et al., 2007; Meer et al., 2014) and humans (Wimmer and Shohamy, 2012). An age-related decline in the ability to flexibly control attention, as measured in our study, has been speculated to impair such integration of information (Hasher and Zacks, 1988; Gazzaley, 2013). Accordingly, we found cognitive control ability to be directly correlated with signals in regions of the episodic-valuation-network including the hippocampus, the ACC and the ventral striatum when delayed options were combined with episodic events. The ACC as well as the ventral striatum are involved in the filtering and controlling of competing and goal-relevant information (Rushworth, 2008; Demanet et al., 2013; Haeger et al., 2015). Moreover, the ventral striatum has been implicated in the structuring of memory encoding and retrieval based on the expected utility of memories (reviewed by Scimeca and Badre, 2012).

The present data provide empirical evidence for recently postulated assumptions from the “Affect-integration-motivation-framework” (Samanez-Larkin and Knutson, 2015). The authors suggest that while reward anticipation may be preserved in aging, the phase where memory content and other information have to be integrated in the decision-process might critically rely on fluid cognitive abilities. In our study, when delayed reward options were combined with episodic events, value computation and future thinking might have strongly competed for attentional resources in older participants with lower control ability. The Singleton task provides a primary measurement of distraction control, which nicely fits with such interpretation. Alternatively, we cannot rule that other functions that are typically also engaged in these tasks may play a critical role here. For example, working memory capacity has previously been associated with temporal discounting in young adults (Hinson et al., 2003; Shamosh et al., 2008). Both functions and their specific effects should be separately assessed in future studies.

A critical impact of cognitive functioning has already been demonstrated in the context of standard delay discounting where episodic prospection was not specifically controlled (Hinson et al., 2003; Shamosh et al., 2008; Huckans et al., 2011; Boyle et al., 2012; Halfmann et al., 2013; Lindbergh et al., 2014; James et al., 2015). Yet it is likely that a certain degree of episodic future thinking is also induced in standard discounting tasks (Mitchell et al., 2011; Peters, 2011). In our design, participants were explicitly instructed to either avoid imagination of specific events (control condition) or to systematically apply episodic prospection (episodic condition) and that we were interested in decision making under different conditions (similar to Peters and Büchel, 2010; Sasse et al., 2015). Given the observed behavior during the training phase, significant activation of episodic neuro-circuits during the tag-conditions as well as participants’ answers during post-scan interviews, it seems unlikely that our results were influenced by difficulties to follow the task. Although we cannot rule out that participants also engaged in episodic prospection in the control condition, our data argue that only under conditions of increased episodic simulation, value integration capacity seemed to be overcharged among older adults with lower cognitive control ability.

In line with previous findings (Viard et al., 2011), most regions of the episodic network reported in younger age were more activated during the episodic compared with the control condition in our study, indicating that older participants engaged imagination. Yet, some key nodes, including the lateral parietal cortex and the hippocampus, did not show differential activation (Peters and Büchel, 2010; Sasse et al., 2015). In addition, compared with data from a younger sample published previously (Sasse et al., 2015), older adults reported fewer imagined internal details for the episodic events. Age-related impairments in the construction and elaboration of episodic simulations have been discussed before (Addis et al., 2011; Schacter et al., 2013) and probably depend on age-specific changes in underlying neural networks (Nyberg et al., 2012). Similar to our previous study (Sasse et al., 2015), episodic prospection capacity did not explain substantial variance in delay discounting in older participants. This could indicate that in our specific paradigm, in which only four rather similar events had to be imagined, a certain degree of imagination can already trigger the tag-effect, if integrated into value computation. Previous work that could reveal a direct relationship between the tag-effect and the vividness of imagined events (Peters and Büchel, 2010) was based on real subject-specific episodic events which might have generated more systematic variability. In addition, the lacking correlation might be caused by the limited sensitivity of our post-scan interview on imagination. Future studies might be able to provide better indicators of imagination quality by assessing trial-wise imagination scores during the task without influencing behavior in the primary intertemporal choice task and by using individual real events.

It is important to note that our findings are limited to older adults and no direct conclusion can be drawn with respect to the impact of cognitive control on discounting behavior in young age, since cognitive functioning was not assessed in our previously published and independent study in younger adults (Sasse et al., 2015). Given that cognitive control is affected by age-related decline, however, we think that our data make an important contribution to understanding the high variability previously observed in discounting studies with older adults and underline the importance of including general cognitive markers in studies addressing complex decision behavior in aging.

Our rather small sample size did not allow for group-wise analyses of neurobehavioral findings, which is a limitation of this study. The specific findings in our very homogenous sample nevertheless strongly argue for a critical consideration of cognitive task demands and executive functioning when studying temporal discounting in late-life. Improving future choice behavior by stimulating episodic prospection (“tag-effect”) has been discussed as therapeutic intervention in patients with characteristic impulsivity (Wiehler and Peters, 2015). Our findings suggest that this benefit may not apply to older people with lower levels of cognitive control ability. Recent data in aged rats and humans have demonstrated beneficial effects of specific adaptive trainings on attentional control ability and its neural correlates (Mishra et al., 2014). It would be interesting to see whether such effects also generalize to performance in higher order tasks like temporal discounting.

## Author Contributions

SB and JP developed the study design. SB, JP and LKS jointly designed the experiment. LKS programmed the paradigm and she collected and processed all data. SB and LKS prepared the manuscript.

## Funding

The study was supported by the German Research Foundation (DFG, grant BR 2877/2-2 to SB and PE 1627/3-1 to JP).

## Conflict of Interest Statement

The authors declare that the research was conducted in the absence of any commercial or financial relationships that could be construed as a potential conflict of interest.
